# Human *Rickettsia heilongjiangensis* Infection, Japan

**DOI:** 10.3201/eid1608.100049

**Published:** 2010-08

**Authors:** Shuji Ando, Masahiro Kurosawa, Akiko Sakata, Hiromi Fujita, Katsurou Sakai, Masao Sekine, Masanori Katsumi, Wakana Saitou, Yasuhiro Yano, Nobuhiro Takada, Ai Takano, Hiroki Kawabata, Nozomu Hanaoka, Haruo Watanabe, Ichiro Kurane, Toshio Kishimoto

**Affiliations:** National Institute of Infectious Diseases, Tokyo, Japan (S. Ando, A. Sakata, A. Takano, H. Kawabata, N. Hanaoka, H. Watanabe, I. Kurane, T. Kishimoto); Sendai Medical Center, Sendai, Japan (M. Kurosawa, W. Saitou); Ohara General Hospital, Fukushima, Japan (H. Fujita); Sendai City Institute of Public Health, Sendai (K. Sakai, M. Sekine, M. Katsumi); Fukui University, Fukui, Japan (Y. Yano, N. Takada); Gifu University, Gifu, Japan (A. Takano, H. Kawabata, H. Watanabe); 1 These authors contributed equally to this article.

**Keywords:** vector-borne infections, Rickettsia heilongjiangensis, Rickettsia japonica, Haemaphysalis concinna, ticks, spotted fever group rickettsiae, bacteria, Japan, dispatch

## Abstract

A case of *Rickettsia heilongjiangensis* infection in Japan was identified in a 35-year-old man who had rash, fever, and eschars. Serum contained *R. heilongjiangensis* antibodies, and eschars contained *R. heilongjiangensis* DNA. *R. heilongjiangensis* was also isolated from ticks in the suspected geographic area of infection.

Spotted fever group (SFG) rickettsiosis is the most prevalent arthropod-borne infectious disease in Japan (*1*). Before publication of a 1984 report about Japanese spotted fever (JSF) caused by *Rickettsia japonica*, scrub typhus caused by *Orientia tsutsugamushi* had been known as the sole rickettsiosis in Japan (*1*). Although many SFG *Rickettsia* species (*R. japonica*, *R. helvetica*, *R. tamurae*, *R. asiatica*, and other related *Rickettsia* spp.) were known, only *R. japonica* had been isolated or detected by PCR from Japanese SFG rickettsiosis patients (*1**–**3*). *R. japonica* was found in *Dermacentor taiwanensis*, *Haemaphysalis cornigera*, *H. flava*, *H. formonensis*, *H. hystricis*, *H. longicornis*, and *Ixodes ovatus* ticks, and *R. helvetica* in *H. japonica*, *I. columnae*, *I. monospinosus*, *I. ovatus*, *I. pavlovskyi*, *I. persulcatus*, and *I. turdus* ticks (*3**,**4*). Cases of SFG rickettsiosis caused by *R. heilongjiangensis*, showing mild rash associated with fever and an eschar, have been reported in the Russian Far East and the People’s Republic of China (*5**–**8*). In Russia and China, *R. heilongjiangensis* was isolated from *H. concinna* and *D. sylvarum* ticks (*6**,**7*). Highly related *Rickettsia* spp. were detected from *H. longicornis* ticks by PCR in South Korea (*9*). In this study, we confirmed a human case of *R. heilongjiangensis* infection in Japan. We also isolated *R. heilongjiangensis* from *H. concinna* ticks, a probable transmission vector, in the suspected geographic area of infection.

## The Study

A 35-year-old man had chills and malaise on July 29, 2008 (day 0). On day 3, the patient became febrile (39.3°C). On day 5, a physician recognized the rash and prescribed oral minocycline (200 mg/d). On day 6, the patient was hospitalized because of constant fever and a whole body rash of unknown cause. At that time, laboratory data showed leukocyte count 7.2 × 10^9^ cells/L, thrombocyte count 275 × 10^9^ cells/L, aspartate aminotransferase 129 U/L, alanine aminotransferase 98 IU/L, and C-reactive protein 3.5 mg/dL. Biopsies were performed on eschars 1 and 2 (5–8 mm diameter) with erythema (≈20 mm diameter), above the left scapula and on the right lower back. During hospitalization, the patient received minocycline, 200 mg/day, intravenously. DNA was extracted from skin biopsy specimens by using a commercial kit according to the manufacturer’s instructions (Gentra Puregene; QIAGEN, Valencia, CA, USA). PCR was performed by using primers of 3 rickettsial genes: outer membrane protein A (*ompA*; primers Rr190.70p and Rr190.602n) (*10*), citrate synthase (*gltA*; primers Cs2d and CsEndr) (*6*), genus *Rickettsia*–specific outer membrane (17-kDa antigen gene; primers R1 and R2) (*11*), and primers for *O. tsutsugamushi*, as reported previously (*12*).

**Figure 1:**
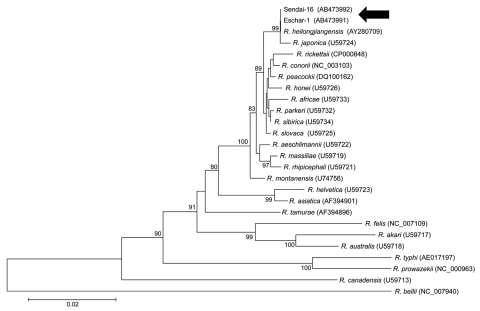
Phylogenetic analysis of citrate synthase (*gltA*) sequences of *Rickettsia* spp. Sequences were aligned by using MEGA4 software (www.megasoftware.net). Neighbor-joining phylogenetic tree construction and bootstrap analyses were performed according to the Kimura 2-parameter distances method. Pairwise alignments and multiple alignments were performed with an open gap penalty of 15 and a gap extension penalty of 6.66. The percentage of replicate trees in which the associated taxa were clustered together in the bootstrap test (1,000 replicates) was calculated. Phylogenetic branches were supported by bootstrap values of >80%. All positions containing alignment gaps and missing data were eliminated in pairwise sequence comparisons (pairwise deletion). Scale bar indicates the percentage of sequence divergence. Arrows indicate eschar specimens.

Although many cases of *R. heilongjiangensis* infection show a single eschar as a result of a tick bite, *ompA*, *gltA*, and 17-kDa antigen genes were detected by PCR (but not with *O. tsutsugamushi*–specific primers) in both eschar specimens. Amplicons were sequenced and analyzed phylogenetically (Figure 1). The 491-bp fragment of *ompA* from eschar 1 (GenBank accession no. AB473995) demonstrated 99.8% and 97.1% nucleotide homology with *R. heilongjiangensis* strain HLJ-054 and *R. japonica* strain YM, respectively. The 1,250-bp fragment of *gltA* of eschar 1 (accession no. AB473991) demonstrated 99.9%, 99.8%, and 96.8% nucleotide homology with the *R. heilongjiangensis* strain HLJ-054, *R. japonica* strain YM, and *R. helvetica* strain C9P9, respectively. The 392-bp fragment of the 17-kDa antigen gene of eschar 1 (accession no. AB473987) demonstrated 100.0% and 99.2% nucleotide homology with *R. heilongjiangensis* strain HLJ-054 and *R. japonica* strain YM, respectively. Blood specimens were negative for rickettsial antigens by PCR, possibly because they were collected after minocycline treatment. Three serial blood samples were tested serologically by immunoperoxidase assays against rickettsial antigens: *R. japonica* strain YH; *O. tsutsugamushi* Karp, Kato, Gilliam, Kawasaki, Kuroki, and Shimokoshi strains; and *R. heilongjiangensis* strain CH8–1 (*13*). The *R. heilongjiangensis* strain CH8-1 used in our analysis was isolated from *H. concinna* ticks collected in Inner Mongolia, China, as an unknown SFG *Rickettsia* species (*3*). Strain CH8–1 was identified as *R. heilongjiangensis* by DNA analysis in this study (*ompA*, accession no. AB473813; *gltA*, AB473812; and 17-kDa antigen genes, AB473811). *R. japonica* and *R. heilongjiangensis* antibody titers were substantially elevated from day 6 to day 16. Titers against *R. heilongjiangensis* were 2–4 times higher than titers against *R. japonica* on day 16 (Table).

**Table:** Antibody titers to spotted fever group rickettsiae in patient’s serum samples, Sendai, Japan, 2008*

Days after symptom onset	Antibody titers (IgG/IgM)		
	*Rickettsia japonica*	*R. heilongiangensis*	*Orientia tsutsugamushi*
6	<10/<10	<10/<10	<10/<10
16	40/160	160/320	<10/<10
23	320/640	320/640	<10/<10

*Ig, immunoglobulin.

**Figure 2:**
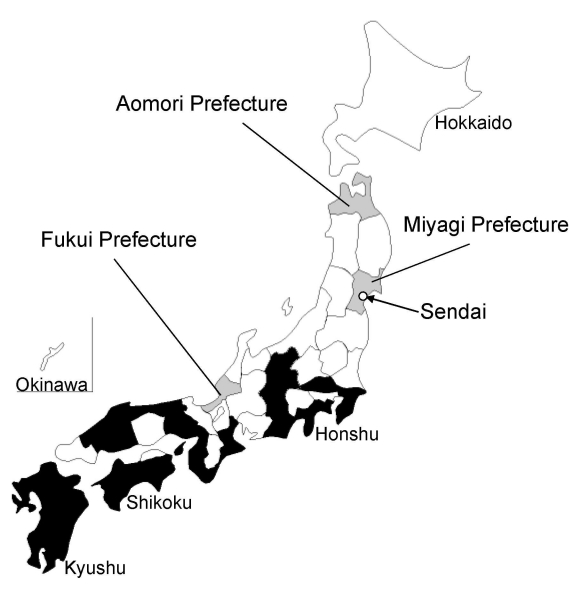
Distribution of reported Japanese spotted fever cases in Japan (≈2008). Prefectures in which Japanese spotted fever cases were reported up to 2008 are shown in black; Fukui, Aomori, and Miyagi prefectures are shown in gray. The map was drawn by using data on reported infectious diseases in Japan (http://idsc.nih.go.jp/idwr/pdf-j.html).

An interview with the patient after the laboratory diagnosis of *R. heilongjiangensis* infection revealed more information about the context of the infection. He resided in an urban area of Sendai, Miyagi Prefecture, Japan (Figure 2). For 2 weeks before onset of symptoms, his outdoor activity was limited to daily walking with a companion dog along a river near his residence. The suspected area where he may have become infected through a tick bite was investigated in September 2008. We captured and examined 72 *Haemaphysalis* spp. ticks (52 *H. longicornis*, 15 *H. concinna*, 4 *H. flava*, and 1 *H. megaspinosa*) and 7 rodents (4 *Rattus norvegicus* and 3 *Microtus montebelli*) for investigation of SFG *Rickettsia* spp. Tick and rodent spleens were homogenized and subjected to isolation studies with L929 cells in shell vial (*3*), and detection of *Rickettsia* DNA by PCR was performed in parallel as previously described. Of the 72 tick samples, 3 *H. concinna* nymphs yielded *Rickettsia* isolates and a DNA fragment of *Rickettsia*, which was detected by PCR. Sequences of 3 isolates and amplicons were identical to those from the patient’s specimens (Figure 1, tick-derived isolates assigned Sendai-16, 29, 32; Sendai-16: *ompA*, *gltA*, and 17-kDa antigen gene accession nos. AB473996, AB473992, and AB473988, respectively; Sendai-29: *ompA*, *gltA*, and 17-kDa antigen gene accession nos. AB473997, AB473993, and AB473989, respectively; and Sendai-32: *ompA*, *gltA*, and 17-kDa antigen gene accession nos. AB473998, AB473994, and AB473990, respectively). PCR-detectable rickettsial agents were not isolated from the rodents; however, 3 of the 4 *R. norvegicus* specimens had high antibody titers to *R. heilongjiangensis*.

To date, most cases of SFG rickettsiosis have been reported as JSF in the western regions of Honshu Island, Japan (*1**,**2*). *R. japonica* was isolated from ixodids in the area where JSF is endemic, and *R. helvetica* from the entire country of Japan (*3*). Moreover, only *R. japonica* has been isolated from patients with SFG rickettsiosis (*1**,**2*). A case-patient with JSF demonstrated serologic evidence of SFG rickettsiosis caused by agents other than *R. japonica*; however, those agents have not been defined (*R. helvetica* in Fukui) (*14*). In 2007, another case of JSF was detected serologically by using only *R. japonica* antigen in Aomori Prefecture, the northernmost prefecture of Honshu Island (*15*). However, *R. japonica* has not been detected in this area (*3*). These results suggest that some cases of SFG rickettsiosis in Japan may have been caused by SFG *Rickettsia* species other than *R. japonica*.

The case reported in this article occurred in an urban area of Sendai in the northern section of Honshu Island (Figure 2). Scrub typhus caused by *O. tsutsugamushi* occurs in this area with 2 seasonal peaks: from early spring to early summer, and from early fall to early winter (*1*). Serologic and microbiologic data ruled out scrub typhus in the present case. *R. heilongjiangensis* infection has been reported in the summer in the disease-endemic area of the Eurasian continent. Notably, the present case occurred in midsummer.

## Conclusions

*R. japonica* has been the only known causative agent of SFG rickettsiosis in Japan, possibly because of limited availability of laboratory test systems. Further studies are needed to define the prevalence of SFG rickettsiosis caused by *Rickettsia* species other than *R. japonica*.
